# Hierarchical AuNPs-Loaded Fe_3_O_4_/Polymers Nanocomposites Constructed by Electrospinning with Enhanced and Magnetically Recyclable Catalytic Capacities

**DOI:** 10.3390/nano7100317

**Published:** 2017-10-12

**Authors:** Rong Guo, Tifeng Jiao, Ruirui Xing, Yan Chen, Wanchun Guo, Jingxin Zhou, Lexin Zhang, Qiuming Peng

**Affiliations:** 1 State Key Laboratory of Metastable Materials Science and Technology, Yanshan University, Qinhuangdao 066004, China; guorong@stumail.ysu.edu.cn (R.G.); pengqiuming@ysu.edu.cn (Q.P.); 2 Hebei Key Laboratory of Applied Chemistry, School of Environmental and Chemical Engineering, Yanshan University, Qinhuangdao 066004, China; rrxing@ipe.ac.cn (R.X.); chenyan@ysu.edu.cn (Y.C.); wc-g@ysu.edu.cn (W.G.); zhanglexin@ysu.edu.cn (L.Z.); 3 State Key Laboratory of Biochemical Engineering, Institute of Process Engineering, Chinese Academy of Sciences, Beijing 100190, China

**Keywords:** Au nanoparticles, composite materials, catalytic reduction, electrospinning, p-nitrophenol

## Abstract

Gold nanoparticles (AuNPs) have attracted widespread attention for their excellent catalytic activity, as well as their unusual physical and chemical properties. The main challenges come from the agglomeration and time-consuming separation of gold nanoparticles, which have greatly baffled the development and application in liquid phase selective reduction. To solve these problems, we propose the preparation of polyvinyl alcohol(PVA)/poly(acrylic acid)(PAA)/Fe_3_O_4_ nanocomposites with loaded AuNPs. The obtained PVA/PAA/Fe_3_O_4_ composite membrane by electrospinning demonstrated high structural stability, a large specific surface area, and more active sites, which is conducive to promoting good dispersion of AuNPs on membrane surfaces. The subsequently prepared PVA/PAA/Fe_3_O_4_@AuNPs nanocomposites exhibited satisfactory nanostructures, robust thermal stability, and a favorable magnetic response for recycling. In addition, the PVA/PAA/Fe_3_O_4_@AuNPs nanocomposites showed a remarkable catalytic capacity in the catalytic reduction of p-nitrophenol and 2-nitroaniline solutions. In addition, the regeneration studies toward p-nitrophenol for different consecutive cycles demonstrate that the as-prepared PVA/PAA/Fe_3_O_4_@AuNPs nanocomposites have outstanding stability and recycling in catalytic reduction.

## 1. Introduction

Au has long been considered to be invaluable precious metals; this did not change until 1973, when Bond et al. revealed the potential application of small-sized Au in hydrogenation reactions [1]. Haruta and Hutchigns et al. in 1987 found that Au nanoparticle catalysts with a size of about 5 nm have good activity in catalyzing oxidation of CO and the reaction of acetylene to vinyl chloride, respectively [2,3]. After that, more and more attention was paid to the nanoscale Au catalysts due to their unusual physical and chemical properties for a variety of catalytic reactions [4,5,6,7,8]. Moreover, in addition to the excellent performances in CO low-temperature oxidation [9,10,11], the epoxidation of propylene [12,13,14], and water gas shift reactions [15,16,17], Au nanoparticles (AuNPs) show outstanding catalytic ability in liquid phase selective oxidation [18,19,20] and selective reduction [21,22,23]. However, the development of applications in liquid phase selective reduction of AuNPs catalysts have been critically restricted because the massive agglomeration of AuNPs results from their high surface energy and strong van der Waals attraction [24], so the catalytic activity shows a foreseeable sharp decrease in the liquid selective catalytic reduction system. In addition, the significant disadvantages of nanoscale AuNPs are their time-consuming separation [25], which provides an obstruction to facile catalyst recovery and recycling. Once the AuNPs catalyst is applied to industrial practical applications, the separation of AuNPs from the catalytic reaction system requires a faster approach. Considering the above problems, AuNPs immobilization on solid supports is regarded as a conventional and feasible method [26,27,28,29,30,31]. Chairam et al. synthesized mung bean starch-AuNPs composite, which acted as both the reducing and stabilizing agents [32]. Zhu et al. immobilized AuNPs on a 2D graphene oxide/SiO_2_ hybrid, showing excellent dispersion and catalytic performance [33]. Kuroda et al. directly deposited AuNPs on poly(methyl methacrylate) beads and the average diameter was 6.9 nm [34]. Zhang et al. obtained Au nanostructures/GO nanocomposites, also exhibiting good catalytic activity by using tannic acid as a reducing and immobilizing agent [35]. Ye et al. synthesized reduced graphene oxide wrapped by polydopamine on which the Pt–Au dendrimer-like nanoparticles were loaded [36]. The nanocomposites exhibit higher catalytic activity, which is substantially affected by Pt-to-Au molar ratios and a superior efficiency for the purification of water containing 4-nitrophenol. Jin et al. coated conducting polymer polyaniline (PANI) on SiO_2_ templates assembled by Fe_3_O_4_ and Au nanoparticles and fabricated Au@Fe_3_O_4_@PANI hybrid shells followed by the removal of the SiO_2_ template [37]. This structure has high stability, recyclability, and largely improves the catalytic activity toward the reduction of 4-nitrophenol. 

On the other hand, electrospinning technology can produce continuous fibers with micro/nanoscale diameters, which have drawn wide interest in recent decades by using a suspended droplet of polymer solution or melt at high voltage [38,39,40]. The electrospun fibers have many outstanding merits, such as good specific surface area [41,42], favorable porosity [43], and great flexibility [44,45], as well as remarkable controllable thickness and diverse architecture [46,47]. Therefore, on the basis of the research of solid supports and many interesting advantages of electrospun fibers, we devote our effort to solving the agglomeration and separation of AuNPs on the premise of guaranteeing small size and high activity. The as-prepared PVA/PAA/Fe_3_O_4_ membranes were neatly synthesized by taking advantage of electrospinning, while the in situ Au nanoparticles from the HAuCl_4_ and NaBH_4_ solution are firmly immobilized on the surface of the nanofibers with the aid of hydrogen bonds. Better specific surface areas and more active sites in the obtained electrospinning membrane promote better dispersion of AuNPs on the surface of the membranes. Thus, the possibility of agglomeration of AuNPs is enormously declined and the stability of catalysts during the catalytic reduction process is constantly in good condition. In addition, Fe_3_O_4_ nanoparticles can contribute to the magnetic recyclability of the nanocomposite membrane in the liquid reaction system, which seems helpful in terms of solving the problems of separation and recovery of the PVA/PAA/Fe_3_O_4_@AuNPs catalyst. Moreover, the preparation process of solid supports via electrospinning is highly eco-friendly and easy to operate and regulate, which reflects the dominant position of this nanocomposite in potential large-scale applications of selective catalytic reduction of gold catalysts. Compared to the previous literature summarized in Table 1 [24,32,33,48,49,50,51], our PVA/PAA/Fe_3_O_4_@AuNPs nanocomposites have the advantages of high activity, high stability, and recyclability, which is crucial to the performance evaluation of catalysts. Moreover, presently prepared nanocomposites also have the characteristics of low cost, easy preparation, and environmental friendliness, demonstrating important and potential applications in catalysis fields. 

## 2. Materials and Methods

### 2.1. Materials

Polyvinyl alcohol (PVA, 98–99% hydrolyzed, average M.W. 57,000–66,000), poly(acrylic acid) (PAA, M.W. ~2000) and ferric chloride hexahydrate (FeCl_3_·6H_2_O, 98%) was purchased from Aladdin Reagent (Shanghai, China). Anhydrous sodium acetate was supplied by Guangzhou Guanghua Chemical Reagent Co. Ltd. (Guangzhou, China). Anhydrous ethanol and ethylene glycol was acquired from the Tianjin Guangfu Fine Chemical Research Institute (Tianjin, China). Chloroauric acid tetrahydrate (HAuCl_4_·4H_2_O), sodium borohydride (NaBH_4_), 2-nitroaniline (2-NA), and 4-nitrophenol (4-NP) were purchased from Alfa Aesar (Beijing, China). Ultra-pure water was obtained through a Milli-Q Millipore filter system (Millipore Co., Bedford, MA, USA) with a resistivity of 18.2 MΩ cm^−1^. All chemicals were used as received without further purification.

### 2.2. Preparation of Electrospun Composites

The 5 g of a 10% aqueous PVA solution was stirred for 8 h at 80 °C. Subsequently, the PVA solution was mixed with 2 g of a 30 wt % aqueous PAA solution, and stirred overnight until the solution was as homogeneous as possible. The volume ratio of the aqueous PVA and PAA solution was 5:2, referring to the previous literature [52]. The Fe_3_O_4_ nanoparticles were prepared according to the reference report [53]. As shown in Figure S1, the diameter of Fe_3_O_4_ nanoparticles range from 200 to 300 nm with a large number of carboxyl groups on the surface. Then, Fe_3_O_4_ nanoparticles (50 mg) were added to a homogeneous aqueous PVA and PAA mixture solution (7 g) and stirred to obtain a well-dispersed solution. The electrospinning precursor solution was held in a 10 mL syringe with the stainless steel needle (20G). During electrospinning, the flow rate was delivered at 0.5 mL·h^−1^, and an aluminum foil was applied as the collector. In addition, the potential difference between the polymer solution and the collector was 20 kV and the distance was 15 cm from the point of needle to collector. After that, the obtained PVA/PAA@Fe_3_O_4_ film sample was dried in a vacuum drying oven at 120 °C for 3 h for heat-induced crosslinking reaction between carboxyl acid groups in PAA and hydroxyl groups in PVA molecules. Aqueous HAuCl_4_ solution (250 µM, 10 mL) and NaBH_4_ aqueous solution (0.01 M, 12 mL) was mixed in a beaker with simultaneous vigorous stirring. Apparently, the color of the mixed solution turned red, which means that Au nanoparticles were generated with a pH value of 6.28. Furthermore, excess NaBH_4_ molecules were removed by centrifugation (8000 rpm, 10 min) and washed with ultrapure water three times. PVA/PAA/Fe_3_O_4_ electrospun film was immersed in an AuNPs solution (50 mL) for an hour in room temperature. After that, the PVA/PAA/Fe_3_O_4_@AuNPs nanocomposites were washed by ultrapure water several times and dried and stored at room temperature for further use.

### 2.3. Catalytic Performance Test

**Figure 1:**
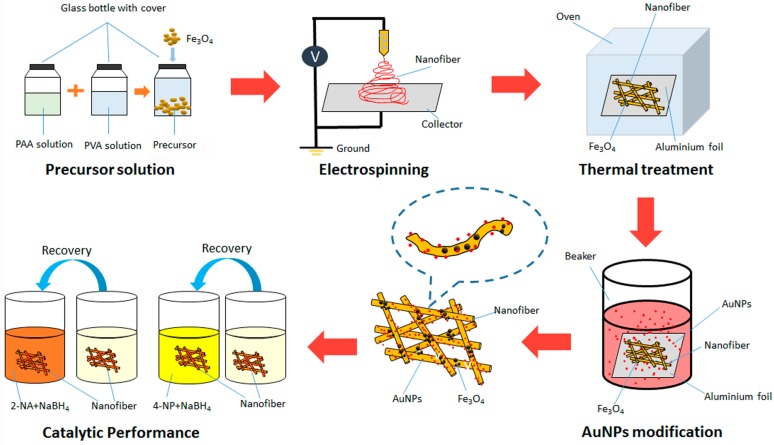
Schematic illustration of the preparation of the PVA/PAA/Fe_3_O_4_@AuNPs composite membrane by electrospinning and its catalytic performance.

The evaluation of catalytic performance of PVA/PAA/Fe_3_O_4_@AuNPs electrospun membrane was executed by catalytic reduction of aqueous 4-NP and 2-NA solution [54]. NaBH_4_ was used as a reducing agent for this catalytic reduction reaction, and all the progress was under the monitoring by UV-VIS spectroscopy at room temperature (Figure 1). The PVA/PAA/Fe_3_O_4_@AuNPs electrospun membrane (10 mg) was added in an aqueous 4-NP solution (10 mL, 0.005 M), followed by adding fresh aqueous NaBH_4_ solution (20 mL, 0.1 M) rapidly. The absorbance was monitored every 3 min by UV-VIS spectroscopy until the solution became colorless. After that, the sample was removed by external magnetic field and washed with ethanol and ultra-pure water for several times. The catalysis of aqueous 2-NA solution (10 mL, 0.005 M) was also applied to evaluate the catalytic capacity of PVA/PAA/Fe_3_O_4_@AuNPs electrospun membrane. In order to further characterize the recycling capacity, the sample catalyzed new aqueous 4-NP and NaBH_4_ mixture solutions 10 times.

### 2.4. Characterization

The microstructure was characterized via scanning electron microscope (SEM) Field Emission Gun FEI QUANTA FEG 250 (FEI Corporate, Hillsboro, OR, USA) with energy dispersive spectroscopy (EDS) for qualitative chemical analysis. All samples have been coated with AuNPs or carbon before SEM measurement. Transmission electron microscopy (TEM, HT7700, High-Technologies Corp., Ibaraki, Japan) was also used to further characterize the obtained samples. High-resolution transmission electron microscopy (HRTEM, Tecnai-G^2^ F30 S-TWIN, Philips, Netherlands) were used to observe the morphologies and microstructures of the samples. X-ray diffraction (XRD) analysis was performed on an X-ray diffractometer equipped with a Cu Kα X-ray radiation source and a Bragg diffraction setup (SMART LAB, Rigaku, Akishima, Japan). Thermogravimetry (TG) characterizations were carried out using a NETZSCH STA 409 PC Luxx simultaneous thermal analyzer (Netzsch Instruments Manufacturing Co, Ltd, Seligenstadt, Germany) in an argon gas atmosphere. FT-IR spectra were obtained by Fourier infrared spectroscopy (Thermo Nicolet Corporation, Madison, WI, USA) via the KBr tablet method. X-ray photoelectron spectroscopy (XPS) was measured on an ESCALAB 250Xi XPS (Thermo Fisher Scientific, San Jose, CA, USA) using 200 W monochromated Al Kα radiation. Both survey scans and individual high-resolution scans for characteristic peaks were recorded. The substrate used for XPS testing is a Si plate purchased from Aladdin Reagent (Shanghai, China). The magnetization was measured by a superconducting quantum interference device (SQUID) magnetometer (MPMS-XL, Quantum Design Inc., San Diego, CA, USA) at 300 K.

## 3. Results and Discussion

### 3.1. Characterization of Nanocomposites

Firstly, Figure 1 illustrates the scheme for the preparation of PVA/PAA/Fe_3_O_4_@AuNPs composite membrane. A high-viscosity polymer solution is the key to the success of electrospinning without considering the influence of voltage and other factors. Here, the use of PVA and PAA for electrospinning is proposed based on the following considerations: The selected PVA and PAA reagents with different molecular weights and volume ratios can well form proper spinning solution with suitable viscosity, concentration, and surface tension. In addition, the crosslinking reaction that occurs between PVA and PAA is effective for further application of the obtained electrospinning membranes. According to Figure 1, the PVA and PAA were dissolved in ultra-pure water and magnetically stirred, and Fe_3_O_4_ nanoparticles were then added. The homogeneous yellow precursor solution was held in a 10 mL syringe with the stainless steel needle (type of 20G) and the PVA/PAA/Fe_3_O_4_ nanocomposites were obtained by electrospinning and dried in a vacuum oven. Due to all of the weighted Fe_3_O_4_ nanoparticles added to the PVA/PAA mixed solution to prepare composite films, we speculated that Fe_3_O_4_ nanoparticles are all in the nanocomposites with complexation efficiency near 100%. After that, PVA/PAA/Fe_3_O_4_ nanocomposite membranes were immersed in a red Au nanoparticle-containing solution. The synthesized AuNPs in aqueous solution have many hydroxyl groups on the surface of particles. In addition, the environment of the AuNPs aqueous solution is neutral, so hydrogen bonds can be expected to form. In addition, there are large numbers of carboxyl groups in the PAA molecules. The nanofiber membranes also have many excess carboxyl groups on the surface. Thus, AuNPs with many hydroxyl groups on the surface can easily load on the surface of prepared nanofibers mainly due to hydrogen bond interaction. The data of Fourier Transform Infrared Spectoscopy (FT-IR) in Figure S2 can also verify the characteristic chemical groups in the obtained composite membranes. The designed PVA/PAA/Fe_3_O_4_@AuNPs nanocomposites were thus obtained.

**Figure 2:**
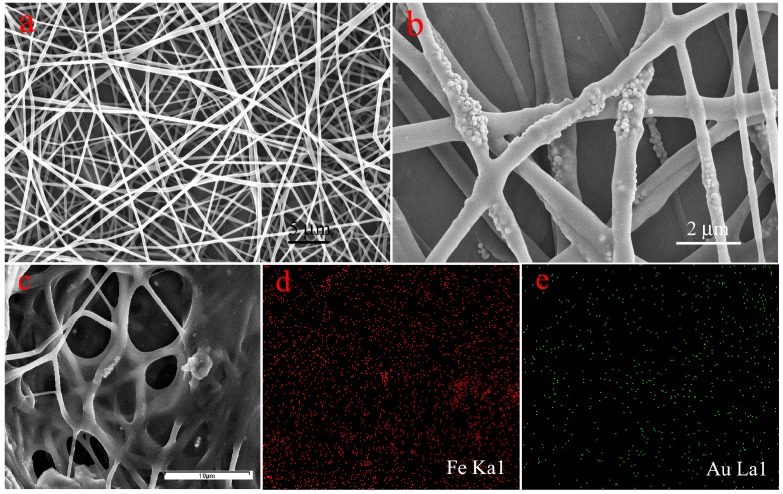
SEM images of the prepared PVA/PAA nanofiber (**a**), PVA/PAA/Fe_3_O_4_ nanofiber (**b**), PVA/PAA/Fe_3_O_4_@AuNPs nanofibers with coated carbon (**c**), and Fe/Au elemental mapping (**d**,**e**).

**Figure 3:**
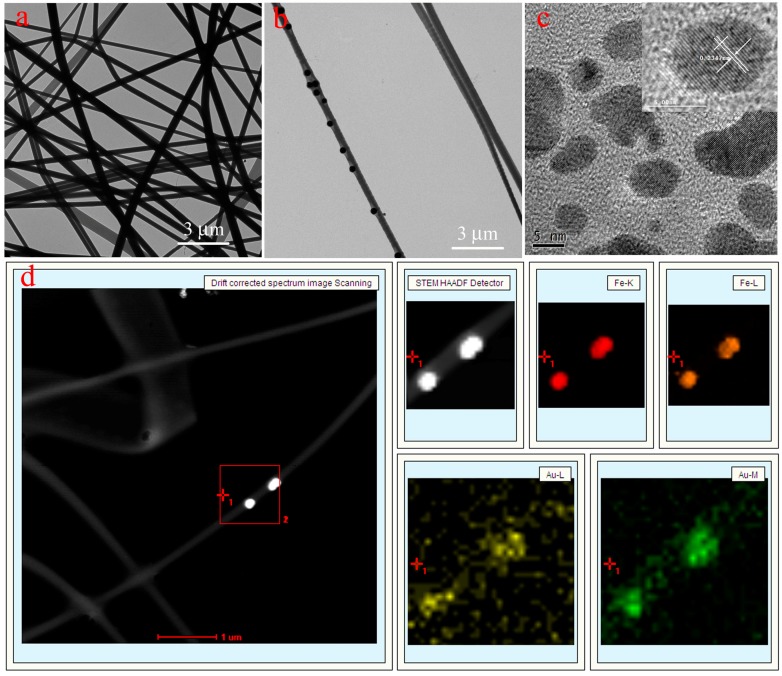
TEM images of the prepared PVA/PAA nanofibers (**a**), PVA/PAA/Fe_3_O_4_ nanofiber (**b**), high resolution of AuNPs (**c**), and PVA/PAA/Fe_3_O_4_@AuNPs nanofibers with Fe/Au elemental mapping (**d**).

Figure 2 depicts the morphology of the obtained nanocomposites. The size and nanostructure of Fe_3_O_4_ nanoparticles can be seen in Figure S1. PVA/PAA nanofibers and PVA/PAA/Fe_3_O_4_ nanofibers have been coated with AuNPs (1–3 nm) before SEM measurement due to organic composites with poor electroconductivity [55,56,57,58,59,60]. While PVA/PAA/Fe_3_O_4_@AuNPs nanofibers have been coated with carbon in order to perform Fe/Au elemental mapping and investigate the presence and localization of Fe_3_O_4_ and AuNPs. The PVA/PAA electrospun fibers present long, straight, and uniform fiber nanostructures with the average diameter of 300 nm according to SEM in Figure 2a. The formed ternary PVA/PAA/Fe_3_O_4_ nanocomposite membranes also have long and straight nanostructures with substantial nanoparticles on the surface and interior space of the fiber, as is shown in Figure 2b. The carboxyl groups on the surface of Fe_3_O_4_ nanoparticles can combine with some hydroxyl groups of PVA molecules. After heat treatment, the prepared fibers became insoluble due to a thermal crosslinking reaction. The diameters of each fiber of PVA/PAA/Fe_3_O_4_ nanocomposites show little differences. In addition, the Fe and Au elemental mapping of PVA/PAA/Fe_3_O_4_@AuNPs nanocomposites have been performed and are shown in Figure 2c–e. We can clearly find that a large number of Fe_3_O_4_ nanoparticles and AuNPs are well distributed onto the obtained composites fibers. In addition, the images of TEM of all samples have been also measured and are shown in Figure 3. Both PVA/PAA fibers and PVA/PAA/Fe_3_O_4_ membranes exhibit long straight fiber nanostructures with Fe_3_O_4_ nanoparticles introduced to the nanofiber skeleton, shown in Figure 3a,b. The diameter of the obtained AuNPs ranges from 5 to 10 nm with a mellow shape [61,62], as shown in Figure 3c. The interplanar spacing of Au nanoparticle is 0.2347 nm, which can well match with the (111) crystal surface of Au. Moreover, the Fe/Au elemental mapping of PVA/PAA/Fe_3_O_4_@AuNPs nanofibers in Figure 3d further confirm the presence and the good distribution of Fe_3_O_4_ and AuNPs in the obtained composite fiber. It can be reasonably speculated that hydrophilic AuNPs successfully load on the surface of PVA/PAA/Fe_3_O_4_ fibers via intermolecular hydrogen bonds, which can be expected to exert catalytic activity and good stability in the next recovery and reuse process.

**Figure 4:**
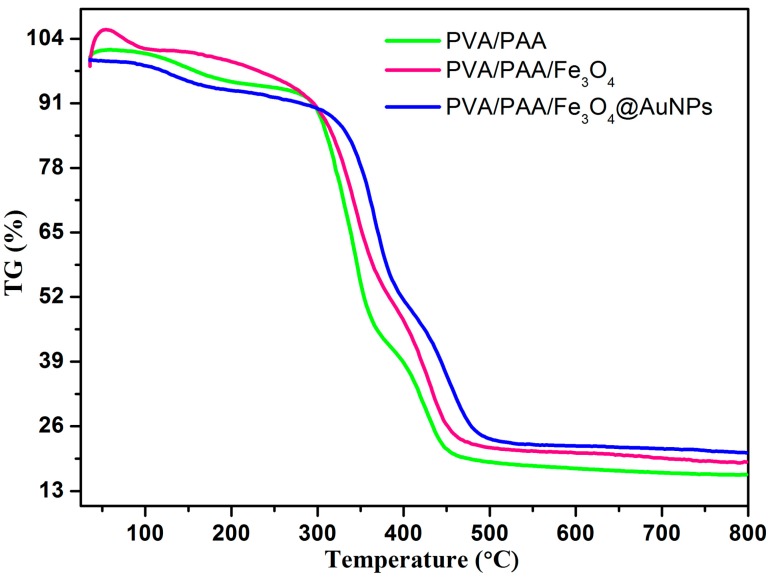
TG curves of PVA/PAA, PVA/PAA/Fe_3_O_4_, and PVA/PAA/Fe_3_O_4_@AuNPs nanocomposites.

Thermogravimetry (TG) curves of samples were measured under an argon atmosphere, as shown in Figure 4. They were performed to measure the thermal stability of the prepared nanocomposites [63,64]. The weight losses below 150 °C can be regarded as the removal of absorbed water, while from 280 to 500 °C, the sharp loss of weight could be attributed to the thermal decomposition of the carbon skeleton in the PVA and PAA molecules. Above 500 °C, the weight values remain stable. Additionally, it was demonstrated that the PVA/PAA/Fe_3_O_4_@AuNPs nanocomposites have better thermal stability. In addition, the weight loss of the PVA/PAA nanofibers was approximately 84.5 wt %, while the PVA/PAA/Fe_3_O_4_ and PVA/PAA/Fe_3_O_4_@AuNPs nanocomposites lost 81.5 and 79 wt %, respectively. The difference in weight loss can be reasonably explained by the incorporation of Fe_3_O_4_ nanoparticles and AuNPs in nanocomposites. 

**Figure 5:**
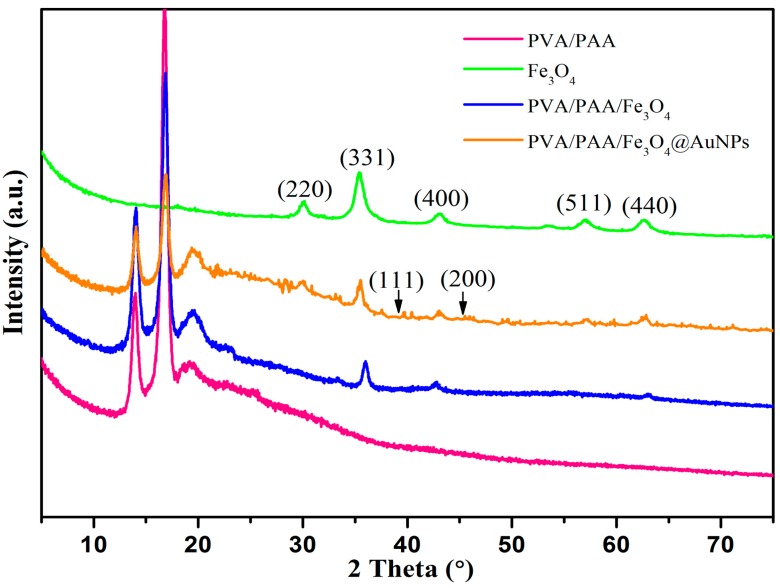
XRD patterns of the obtained PVA/PAA electrospun nanofibers, PVA/PAA/Fe_3_O_4_ nanofibers, PVA/PAA/Fe_3_O_4_@AuNPs nanocomposites, and Fe_3_O_4_ nanoparticles.

XRD data was also measured to further identify the structure of the membrane, as shown in Figure 5. According to the obtained results, the characteristic absorption peaks of 2*θ* at 30.0°, 35.3°, 43.0°, 57.0°, and 62.7° can be assigned to the (220), (311), (400), (511), and (400) planes of the face-centered cubic Fe_3_O_4_ phase. In addition, the PVA/PAA/Fe_3_O_4_ and PVA/PAA/Fe_3_O_4_@AuNPs nanocomposites both have the same characteristic peaks, which indicates the introduction of Fe_3_O_4_ nanoparticles in the nanocomposites. The XRD pattern of the PVA/PAA/Fe_3_O_4_@AuNPs nanocomposite, compared to the XRD patterns of the PVA/PAA nanofibers and PVA/PAA/Fe_3_O_4_ nanocomposites, indicates newly emerging diffraction peaks with 2*θ* values of 38.9° and 46.1°, which are indexed to the (111) and (200) cubic lattice planes of gold nanoparticles. Similar results about diffraction peaks of AuNPs have been reported in previous reports [54,65]. The signals in the XRD measurements of PVA/PAA/Fe_3_O_4_@AuNPs nanocomposites are slightly weak mainly due to the thin film state of the nanocomposites containing fewer AuNPs and Fe_3_O_4_ particles in the measurement process.

**Figure 6:**
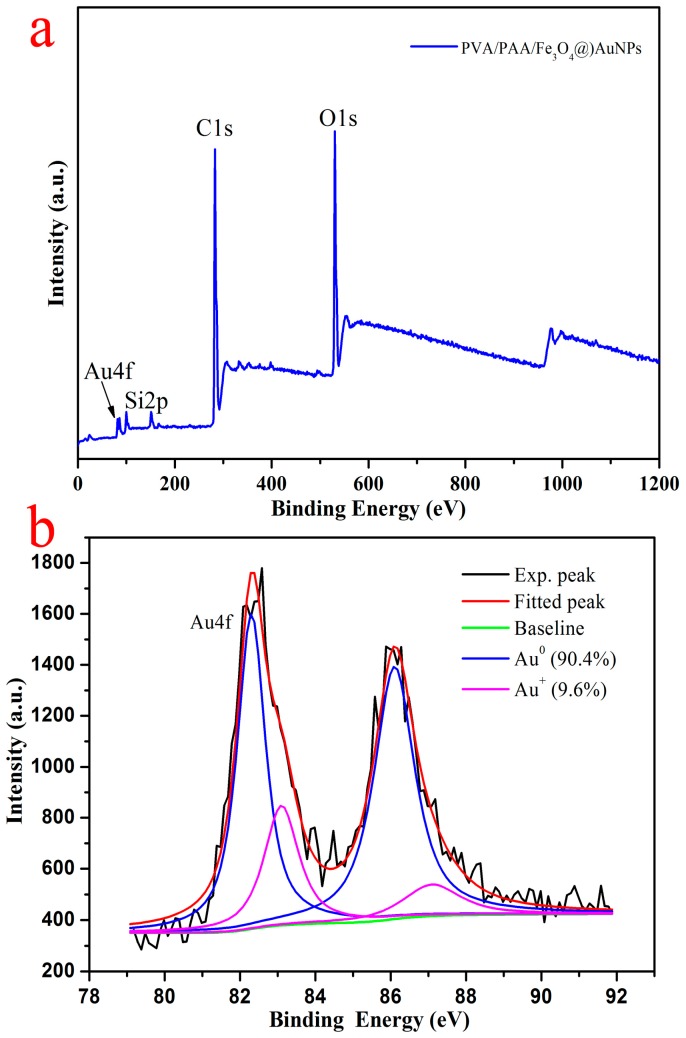
Survey XPS spectra of PVA/PAA/Fe_3_O_4_@AuNPs nanocomposites (**a**) and the deconvolution of XPS peaks of the Au4f region (**b**).

In order to verify the XRD spectra and TG results, the composition analysis of the as-prepared PVA/PAA/Fe_3_O_4_@AuNPs nanocomposite membrane was performed via X-ray photoelectron spectroscopy (XPS), as shown in Figure 6. The survey data demonstrate the characteristic peaks such as C1s, O1s and Au4f in Figure 6a. The Si2p peak came from the Si plate as a substrate [58,66]. In addition, there is a pair of typical spin splitting peaks of Au4f in the spectra with binding energies of 82.5 and 86.3 eV, and the distance between the two characteristic peaks is 3.8 eV, which can be assigned to the 4f_5/2_ and 4f_7/2_ lines of metallic gold. This is slightly different from the results of a previous study [67] because, in comparison with the main Au^0^ species (accounting for 90.4%), there are only 9.6% Au^+^ species resulting from residual HAuCl_4_ molecules that are not completely restored in situ to Au nanoparticles. The peaks located at around 83.5 and 87.0 eV correspond to the spin orbit splitting components of Au4f. Combined with the above characterization, these results represent the Au^0^ species that have been successfully incorporated on nanofibers.

**Figure 7:**
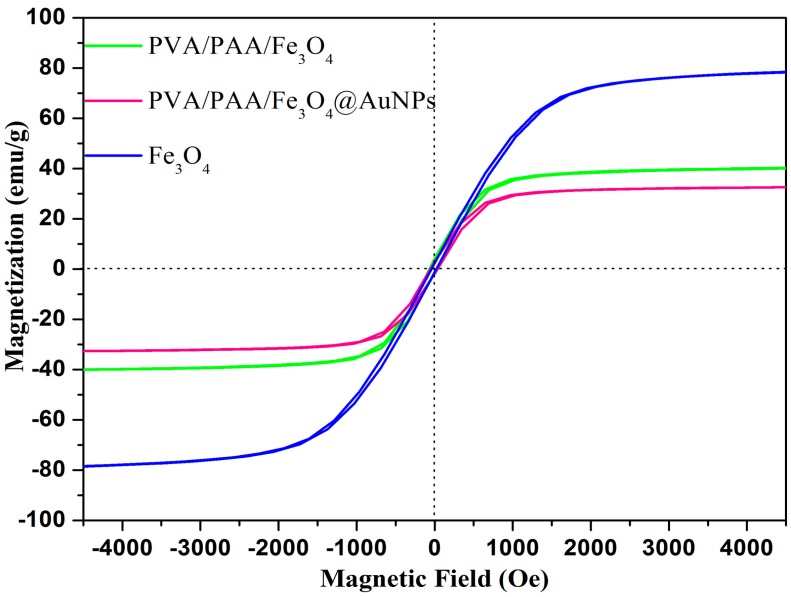
Magnetization hysteresis loops of the obtained PVA/PAA/Fe_3_O_4_ nanocomposites, PVA/PAA/Fe_3_O_4_@AuNPs composites, and Fe_3_O_4_ nanoparticles.

Magnetization hysteresis loops, as shown in Figure 7, are further collected to investigate the magnetic performance. The completely reversible field-dependent magnetization curves mean that all of the samples are super-paramagnetic. The saturation magnetization value of Fe_3_O_4_ nanoparticles, PVA/PAA/Fe_3_O_4_, and PVA/PAA/Fe_3_O_4_@AuNPs nanocomposite membranes are 78.5, 40.0 and 32.5 emu/g, respectively. Due to substantial non-magnetic substance of PVA and PAA molecules as fiber skeleton, the saturation magnetization values have significantly reduced. In addition, compared to the PVA/PAA/Fe_3_O_4_ nanocomposites, the clear decrease in magnetic response indirectly indicates the incorporation of non-magnetic substance AuNPs into the PVA/PAA/Fe_3_O_4_@AuNPs membrane. Although there is obviously a loss of saturation magnetization, this magnetic response can still ensure controllable magnetic recoveries, which shows its great importance in terms of the application of catalytic materials.

### 3.2. Catalytic Reduction Performances

**Figure 8:**
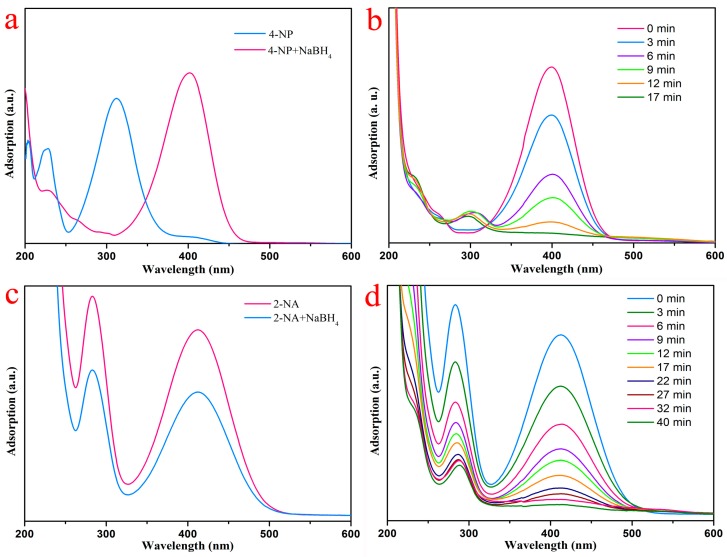
Catalytic reduction of (**a**) 4-NP before and after adding NaBH_4_ aqueous solution; (**b**) reduction of 4-NP with PVA/PAA/Fe_3_O_4_@AuNPs composite; (**c**) 2-NA before and after adding NaBH_4_ aqueous solution; (**d**) reduction of 2-NA with PVA/PAA/Fe_3_O_4_@AuNPs composite.

**Figure 9:**
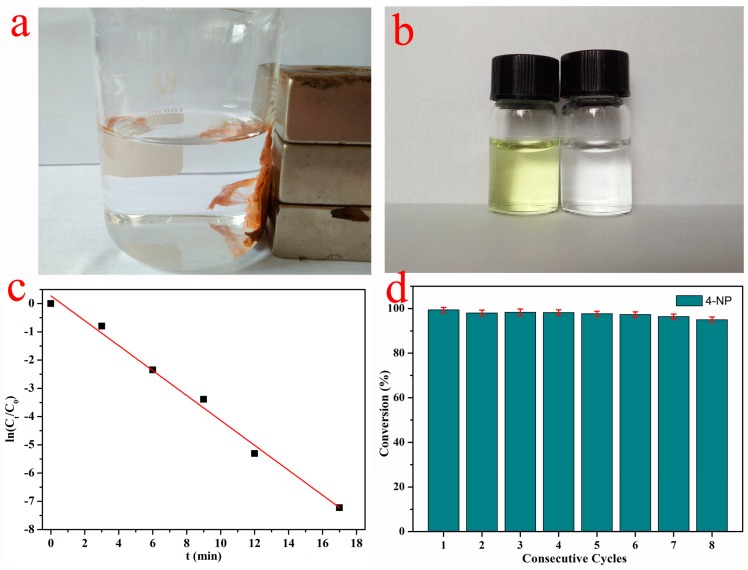
Magnetic recovery of PVA/PAA/Fe_3_O_4_@AuNPs nanocomposites with external magnetic field (**a**); comparison of 4-NP solution before and after catalytic reaction (**b**); the relationship between ln(*C*_t_/*C*_0_) and the reaction time (*t*) of the nanocomposite catalyst (**c**); the reusability test of PVA/PAA/Fe_3_O_4_@AuNPs nanocomposites as catalysts for the reduction of 4-NP (**d**).

The catalytic reduction of 4-NP and 2-NA was carried out to investigate the catalytic activity of the PVA/PAA/Fe_3_O_4_@AuNPs nanocomposite membrane. The 4-NP solution had a strong characteristic peak at 317 nm, as shown in Figure 8a. After the NaBH_4_ solution was added, NaBH_4_ molecules provide negative hydrogen ions to attack 4-NP, and the resultant of the reaction was 4-nitorphenolate. The redshift of the characteristic absorption peak at 402 nm can prove the formation of 4-nitrophenolate. The conversion of 4-NP to the 4-nitrophenolate ion takes place within seconds with the help of excess NaBH_4_ solution, but further reduction does not progress even over 24 h. After the prepared PVA/PAA/Fe_3_O_4_@AuNPs nanocomposites were added, the catalytic reaction began and the time was recorded. Then, with the catalytic reaction of composite materials, the nitro group of 4-nitrophenolate was reduced to amino groups with the catalysis of AuNPs, so the adsorption intensity of 4-nitorphenolate decreased. Thus, the visual performance was the descended sharply of characteristic absorption peaks at 402 nm, as shown in Figure 8b. In addition, it is clear that the bright yellow mixed solution became colorless, as shown in Figure 9b. In addition, the catalytic reduction of the 2-NA solution was also applied to further demonstrate the catalytic activity of nanocomposite membrane. No significant changes of 2-NA solution in the color and characteristic absorption peak at 415 nm were observed before or after adding aqueous NaBH_4_ solution for 24 h, as shown in Figure 8d. After the PVA/PAA/Fe_3_O_4_@AuNPs catalyst was added, the absorption band of 2-NA clearly decreased and the system solution became colorless, which demonstrates that this catalyst also exhibits high catalytic activity. 

**Figure 10:**
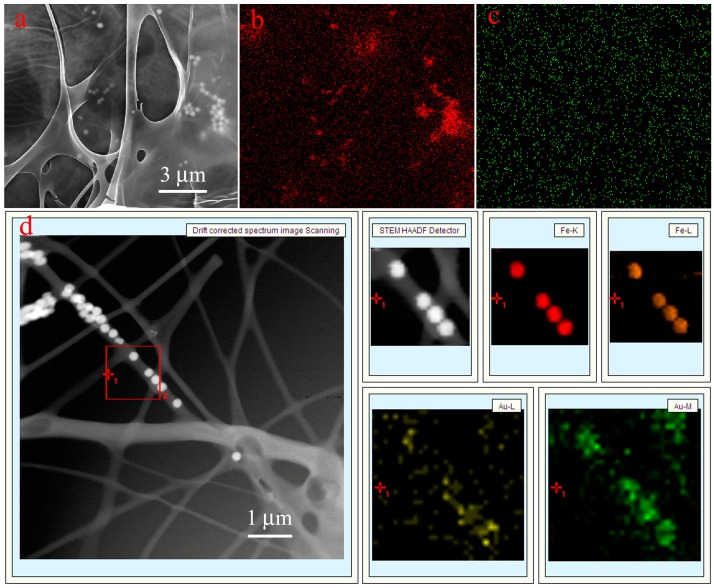
The SEM image (**a**) with Fe/Au elemental mapping (**b**,**c**) and TEM image with Fe/Au elemental mapping (**d**) of PVA/PAA/Fe_3_O_4_@AuNPs nanocomposites after the eighth cycle of catalytic reactions.

In addition, the PVA/PAA/Fe_3_O_4_@AuNPs nanocomposites were easily separated by an external magnetic field (Figure 9a), which also validates the previous magnetic measurements. The reaction of the reduction of 4-NP was assumed to be pseudo-first-order kinetics since the concentration of NaBH_4_ was significantly higher than that of 4-NP and can be considered constant. As shown in Figure 9c, the linear correlation between ln(*C*_t_/*C*_0_) and the reaction time (*t*) confirms the pseudo-first order kinetics of this reaction. *C*_t_ and *C*_0_ are the concentrations of 4-NP at time t and the time of the initial concentration, respectively. The pseudo-first-order reaction rate constant (k) was calculated to be 0.441 min^−1^ for the reduction of 4-NP. In order to further study the stability and catalytic activity of the PVA/PAA/Fe_3_O_4_@AuNPs catalyst, the nanocomposites were allowed to continuously proceed to catalyze a fresh 4-NP and NaBH_4_ system eight times to evaluate the recyclable properties, as summarized in Figure 9d. As expected, after eight reductions of 4-NP, the conversion still maintained high catalytic activity and reached a value of 92%. Compared to the first reduction process, the slight decrements of conversion demonstrate excellent stability of PVA/PAA/Fe_3_O_4_@AuNPs composite membrane. In addition, the SEM and TEM images with Fe/Au elemental mapping of nanocomposites after the eighth cycle of catalytic reactions are also demonstrated in Figure 10. It can be easily observed that the PVA/PAA/Fe_3_O_4_@AuNPs nanocomposites can basically retain the original nanostructure, demonstrating that the prepared composite materials are remarkably stable. After repeated washing and drying in the reuse process, the slightly deformed membrane composite materials still maintained high catalytic performances. Moreover, Fe_3_O_4_ nanoparticles still firmly immobilize inside the membrane, which guarantees magnetic performance and recyclability. Thus, the prepared nanocomposites have outstanding stability and demonstrate great potential application in catalysis fields.

Such a good catalytic performance of the PVA/PAA/Fe_3_O_4_@AuNPs membrane benefits from the use of the electrospun membrane as a support for the gold catalyst. In addition, the loaded AuNPs incorporated on the electrospun PVA/PAA/Fe_3_O_4_ composite membrane show a well-dispersed state, which helps to avoid agglomeration and improve catalytic performances. It should be noted that easy aggregations between AuNPs prevent widespread applications. In recent years, various structures and composites with AuNPs have been designed and investigated, as listed in Table 1. In our present system, the PVA/PAA/Fe_3_O_4_@AuNPs nanocomposites demonstrate nanostructures with an eco-friendly prepared process and superior catalytic properties, as well as magnetically recyclable capacities, suggesting wide catalytic applications.

## 4. Conclusions

The AuNPs-containing PVA/PAA/Fe_3_O_4_ nanocomposite materials were successfully prepared via electrospinning and self-assembly. Au nanoparticles were loaded on the surface of a composite membrane via a self-assembly process. The prepared PVA/PAA/Fe_3_O_4_ nanocomposites provide good support for AuNPs to be loaded on and effectively avoid agglomeration of AuNPs with improved stability for the next catalytic reduction application. In addition, the introduction of magnetic nanoparticles in the present composite catalysts is advantageous to conveniently separate from the reduction solution and reuse for subsequent recycling. For the catalytic reduction of liquid 4-NP and 2-NA solution, the prepared PVA/PAA/Fe_3_O_4_@AuNPs nanocomposite membranes demonstrated significant catalytic activity even after eight cycles for catalytic reduction at room temperature. Thus, the present prepared PVA/PAA/Fe_3_O_4_@AuNPs nanocomposites display excellent catalytic activity, good stability, and outstanding magnetic separation. The present research work thus proposes a novel approach to design and prepare new Au nanoparticle-containing composite materials for applications in selective catalytic reduction.

## Figures and Tables

**Table 1:** Comparative characteristics and catalytic performance of catalyzers in the reported literature.

No.	Catalyzer	Catalytic Performance ln(*C*_t_/*C*_0_) min^−1^	Preparation Method	Characteristics
1	Au@CPF-1 hybrid [24]	0.303	AuNPs synthesized on the activated CPF-1.	Complexed and costly preparation.
2	Starch-supported gold nanoparticles [32]	-	Mix HAuCl_4_ and MBS in DI water.	Weak reducibility of polysaccharides, weak catalytic activity, simple process, and environmentally friendly.
3	Graphene oxide/SiO_2_/AuNPs hybrid nanomaterials [33]	1.04	Graphene oxide/SiO_2_ via a sol–gel process, activated by SnCl_2_, mixed with HAuCl_4_.	Remarkable catalytic capacity, accompanying adsorption process, inconvenient preparation process.
4	TiO_2_/ZnO/AuNF nanofibers [48]	-	Calcined electrospinning nanofibers, SnCl_2_ activated, adding HAuCl_4_ solution.	Toxic solvent in preparation, unfriendly to environment.
5	Fe_3_O_4_@TiO_2_@Ag–Au microspheres [49]	0.1148	3-Aminopropyltrimethoxysilane modified Fe_3_O_4_@TiO_2_ microspheres, Ag nanoparticles replacement, Ag–Au bimetallic nanostructures.	Complexed replacement of Au/Ag, weak catalytic activity.
6	Au/Fe_3_O_4_@hollow TiO_2_ nanoreactor [50]	0.46	AuNPs loaded on magnetic SiO_2_ nanospheres, Fe_3_O_4_ modified, covered with TiO_2_ shell.	Impacted catalytic capacity due to the coverage and isolation of the TiO_2_ shell.
7	Double-shelled sea urchin-like yolk-shell Fe_3_O_4_/TiO_2_/Au microspheres [51]	1.84	Synthesis of Fe_3_O_4_/SiO_2_/TiO_2_ core-shell microspheres by sol–gel process, SiO_2_ shell removed by acid post-treatment, AuNPs loaded.	Remarkable catalytic performance, complexed preparation, negative effect in acid post-treatment.
8	Present work	0.441	AuNPs-loaded, magnetically Fe_3_O_4_ support by electrospinning.	Eco-friendly prepared process, high stability, and good catalytic performance.

## Supplementary Materials

The following are available online at http://www.mdpi.com/2079-4991/7/10/317/s1. Figure S1. SEM (a) with EDX and TEM (b) images of the prepared Fe_3_O_4_ nanoparticles. Figure S2. FT-IR of PVA/PAA, PVA/PAA/Fe_3_O_4_ and PVA/PAA/Fe_3_O_4_@AuNPs nanocomposites.
