# Supplementary material for: Hierarchical AuNPs-Loaded Fe3O4/Polymers Nanocomposites Constructed by Electrospinning with Enhanced and Magnetically Recyclable Catalytic Capacities
Source: Nanomaterials (Basel). 2017 Oct 12;7(10):317. doi: 10.3390/nano7100317 (PMC5666482; doi:10.3390/nano7100317)
Supplement: Supplementary file 1 [file nanomaterials-07-00317-s001.pdf]

Supplementary Information

Hierarchical AuNPs-Loaded Fe_3O_4/Polymers Nanocomposites Constructed by Electrospinning with Enhanced and Magnetically Recyclable Catalytic Capacities

[1] State Key Laboratory of Metastable Materials Science and Technology, Yanshan University, Qinhuangdao 066004, China; guorong@stumail.ysu.edu.cn (R.G.); pengqiuming@ysu.edu.cn (Q.P.)
[2] Hebei Key Laboratory of Applied Chemistry, School of Environmental and Chemical Engineering, Yanshan University, Qinhuangdao 066004, China; rrxing@ipe.ac.cn (R.X.); chenyan@ysu.edu.cn (Y.C.); wc-g@ysu.edu.cn (W.G.); zhanglexin@ysu.edu.cn (L.Z.)
[3] State Key Laboratory of Biochemical Engineering, Institute of Process Engineering, Chinese Academy of Sciences, Beijing 100190, China
* Correspondence: tfjiao@ysu.edu.cn (T.J.); zhoujingxin@ysu.edu.cn (J.Z.); Tel.: +86-335-8056854 (T.J.); +86-335-8061569 (J.Z.)

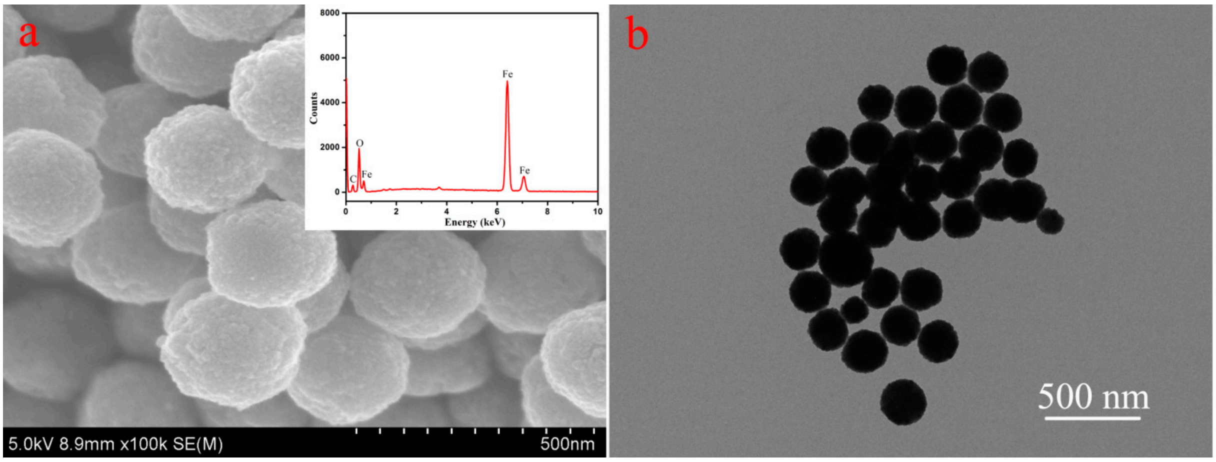

Figure S1. SEM (**a**) with EDX and TEM (**b**) images of the prepared Fe_3O_4 nanoparticles.

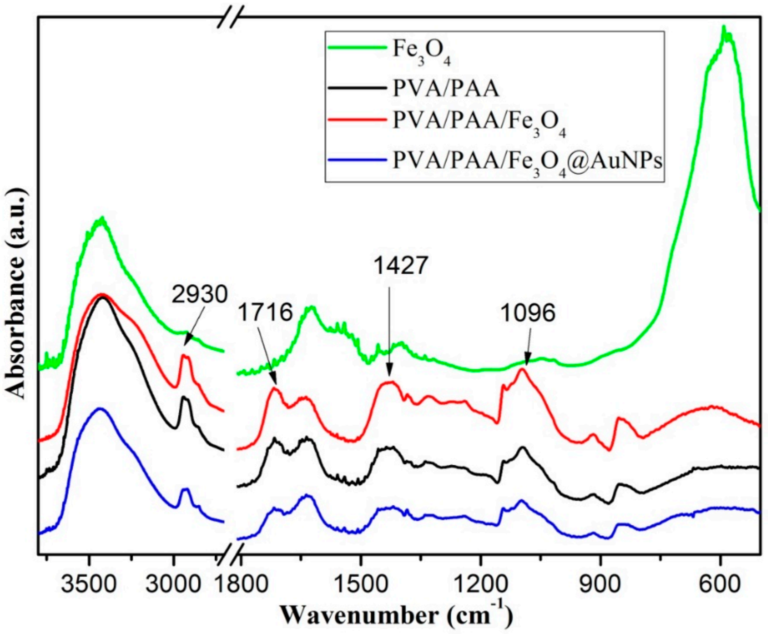

Figure S2. FT-IR of PVA/PAA, PVA/PAA/Fe_3O_4 and PVA/PAA/Fe_3O_4@AuNPs nanocomposites.
